# Occurrence and characterization of methicillin-resistant *Staphylococcus pseudintermedius* in successive parturitions of bitches and their puppies in two kennels in Italy

**DOI:** 10.1186/s12917-018-1612-z

**Published:** 2018-10-11

**Authors:** Michela Corrò, Joakim Skarin, Stefan Börjesson, Ada Rota

**Affiliations:** 1 Istituto Zooprofilattico Sperimentale delle Venezie, viale Università 10, 35020 Legnaro, PD Italy; 2 Department of Microbiology, National Veterinary Institute (SVA), SE-751 89 Uppsala, Sweden; 3 Department of Animal Health and Antimicrobial Strategies, National Veterinary Institute (SVA), SE-751 89 Uppsala, Sweden; 4 Department of Veterinary Sciences, University of Turin, Largo Paolo Braccini 2-5, 10090 Grugliasco, TO Italy

**Keywords:** MRSP colonization, Postpartum bitches, Puppy, Whole-genome sequencing

## Abstract

**Background:**

Multi-drug methicillin-resistant *Staphylococcus pseudintermedius* (MRSP) detection is rapidly increasing in microbial specimens from pets across Europe. MRSP has also been isolated from bitches and newborns in dog breeding kennels. This study assessed whether MRSP lineage differs between breeding kennels and is maintained over time. Post-partum bitches (at day 3 vaginal and day 3, 9 and 35 milk samples) and their litters (at day 3, 9 and 35 oral and abdominal skin samples) from two Italian breeding kennels (A and B) were sampled and MRSP was subsequently characterized via whole-genome sequencing and antibiotic susceptibility testing. The study was carried out from October 2014 to March 2016 and included successive parturitions from the same animals.

**Results:**

The analysis revealed different situations in both investigated kennels. In kennel A, circulating strains were from 7-locus sequence types ST688, ST258 and closely related isolates of ST71, which included most isolates. In kennel B, only a new isolate, ST772, was detected. In addition, most isolates from both kennels had multi-resistant antibiotic profiles. MRSP was only isolated from litters of MRSP-positive bitches, thus suggesting that bitch-litter transmission is likely.

**Conclusions:**

Our data show that MRSP circulation can differ in different settings, that several clonal lineages can circulate together, and that vertical transmission appears common. MRSP colonization did not affect the health conditions of the bitches or of their litters.

**Electronic supplementary material:**

The online version of this article (10.1186/s12917-018-1612-z) contains supplementary material, which is available to authorized users.

## Background

*Staphylococcus pseudintermedius* belongs to the bacterial skin flora of canines and is a common opportunistic pathogen of dogs [1]. In addition, *S. pseudintermedius* is commonly detected in the genital organs of both healthy dogs and dogs with reproductive problems, including in the vaginas of bitches in the pre- and postpartum periods [2, 3]. It has been linked to neonatal mortality due to necrotising dermatitis, pododermatitis, acute suppurative interstitial pneumonia [4], toxaemia and septicaemia [5].

Due to the importance of *S*. *pseudintermedius* as an opportunistic pathogen, it is worrisome that multi-drug methicillin-resistant *S. pseudintermedius* (MRSP) detection has rapidly increased in microbiological specimens from pets across Europe in recent years [6, 7]. MRSP is often regarded as a threat due to limited therapeutic options and because it frequently shows resistance profiles to three or more antimicrobial classes [8, 9]. Among European isolates, the multi-resistant clonal lineage ST71-J-t02-II–III is dominant [10], but other emerging clones, such as ST258, have also been detected in some European countries [11–13]. MRSP has also occasionally been isolated from the colostrum and milk of breeding bitches as well as from puppies [5, 14]. However, data are lacking on the relatedness and transmission of MRSP lineages isolated from bitches and their offspring in breeding kennels. Therefore, this study assessed whether the same MRSP lineage is present in bitches and their litters in different breeding kennels and is maintained over time.

## Methods

### Animals and sampling

Two breeding kennels (A and B) in Piedmont (Italy) were identified as MRSP-positive in a study investigating the microbial flora of bitches and their litters, conducted from October 2014 to March 2016. The current investigation characterized and compared MRSP isolates identified in that study from bitches and their offspring (Table 1).

**Table 1:**
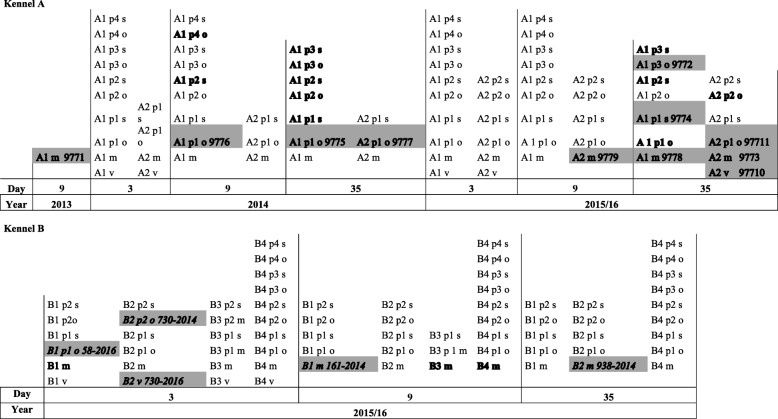
Chronology of isolation of MRSP in two breeding kennels (A, B), from bitches (A1,A2; B1,B2,B3,B4) samples (vagina = v; milk = m) and from mouth (o) or skin (s) samples of their puppies (p), at three sampling times (3, 9 and 35 days post-partum), in the years 2013–2014 and 2015/16. Isolates given in bold are MRSP positive. The genotyped isolates are evidenced in grey and their identification number appears beside

The sampling scheme for the bitches was as follows: vaginal swabs were collected on postpartum day 3, and milk swabs were collected from all mammary glands on postpartum days 3, 9 and 35. The puppies were sampled at the same frequency at two sites: the abdominal skin cranial to the umbilicus (rubbing the swab for 5 s on a 4 cm^2^ area) and the mouth (rubbing the swab on the oral mucosa). All swabs (Copan Italia, Brescia, Italy) were placed in Amies medium (Copan Italia, Brescia, Italy), transported to the laboratory at environmental temperature and cultured within 48 h.

The study was performed in accordance with the guidelines for the care and use of animals of the Department of Veterinary Science of the University of Turin. Informed consent was obtained from the dog breeders prior to testing.

### MRSP identification

The swabs were directly plated onto 5% sheep blood agar (SBA, Oxoid, Basingstoke, UK), for a general assessment of bacterial growth, especially that of staphylococci, and in 6.5% NaCl nutrient broth (Mueller Hinton broth T073–BIOKAR Diagnostic, Allonne, France) and incubated overnight at 37 ± 1 °C. After incubation, 10 μl of the salt broth was inoculated into a selective medium (CHROMagar™ MRSA II plates, Heidelberg, Germany). Staphylococci grown on blood agar plates were presumptively identified based on colony morphology, Gram staining, catalase tests, haemolysis and pigment production. Coagulase-positive staphylococci were identified by tube coagulase testing on rabbit plasma (Istituto Zooprofilattico delle Venezie, Legnaro, Italy) and incubated at 35 °C ± 1 °C for 4 h. One to three isolated colonies were added to 0.5 ml of rabbit plasma in a tube and incubated at 35 °C. Those samples testing negative after 4 h were maintained at room temperature and read again after 18–24 h.

All colonies with pink, light pink or white colouring after 24–48 h on CHROMagar™ MRSA II plates were regarded as suspect methicillin-resistant coagulase-positive staphylococci. Five colonies grown on selective medium plates after enrichment were selected for confirmation of the presence of the *mec*A gene by conventional PCR [15]; staphylococci species identification was also performed by PCR [16].

### Antimicrobial susceptibility tests

Minimum inhibitory concentration (MIC) was determined using broth microdilution (Sensititre COMPAGN1F, Thermo Fisher Scientific, Waltham, MA USA). Isolates were tested for susceptibility to oxacillin and a panel of 18 antimicrobials belonging to nine antimicrobial classes: beta-lactams (penicillin, ampicillin, amoxicillin/clavulanic acid, ticarcillin, ticarcillin/clavulanic acid, cefazolin, cefpodoxime, ceftiofur), tetracyclines (doxycycline), diaminopyrimidine-sulphonamide combinations (trimethoprim/sulfathiazole), aminoglycosides (gentamicin, amikacin), macrolides (erythromycin), lincosamides (clindamycin), fluoroquinolones (enrofloxacin, marbofloxacin), amphenicols (chloramphenicol) and rifamycins (rifampicin).

Antimicrobial susceptibility results were interpreted per the Clinical Laboratory Standards Institute [17, 18]. In case of no clinical breakpoints set for *S. pseudintermedius* in dogs, we used breakpoints for *Staphylococcus* spp. in dogs and, when unavailable in dogs, we used other species (horse and cattle) or human breakpoints (Table 2). For ticarcillin and ticarcillin/clavulanic acid, the [19] were used, and *S. aureus* DSMZ 11729 was used as a quality control.

**Table 2:** Breakpoints for antimicrobial susceptibility of *Staphylococcus pseudintermedius* or *Staphylococcus spp.* according to Clinical and Laboratory Standards Institute (CLSI VET 01 S3, 2015)

Antimicrobial agent				
	S	I	R	
Oxacillin	≤0.25	–	≥0.5	*Staphylococcus pseudintermedius* dog
Ampicillin	≤0.25	–	≥0.5	*S.pseudintermedius* dog
Penicillin	≤0.5	1	≥2.0	*Staphylococcus spp.* (other species: horse)
Amoxicillin/Clavulanic acid	≤0.25/0.12	0.5/0.25	≥1/0.5	*Staphylococcus* spp. dog
Cefazolin	≤2	4	≥8	*S.pseudintermedius* dog
Cefpodoxime	≤2	4	≥8	*S.pseudintermedius* dog
Ceftiofur	≤2	4	≥8	*S. aureus* (other species: cattle)
Trimethoprim-sulfamethox.	≤2/38	–	≥4/76	*Staphylococcus spp.* human-derived MIC breakpoints
Amikacin	≤4	8	≥16	*Staphylococcus spp.* dog
Rifampicin	≤1	2	≥4	*Staphylococcus* spp. human-derived MIC breakpoints
Enrofloxacin	≤0.5	1–2	≥4	*Staphylococcus spp.* dog
Marbofloxacin	≤1	2	≥4	*Staphylococcus spp.* dog
Clindamycin	≤0.5	1–2	≥4	*Staphylococcus spp.* dog
Erythromycin	≤0.5	1–4	≥8	*Staphylococcus* spp. human-derived MIC breakpoints
Doxycycline	≤0.12	0.25	≥0.5	*S. pseudintermedius* dog
Chloramphenicol	≤8	16	≥32	*Staphylococcus spp.* human-derived MIC breakpoints
Gentamicin^a^	≤4	8	≥16	human-derived MIC breakpoints
Ticarcillin^b^	≤16	32–64	≥128	human-derived MIC breakpoints
Ticarcillin/Clavulanic acid^b^	≤16/2	32/2–64/2	≥128/2	human-derived MIC breakpoints

^a^ [18] ^b^ EUCAST, [19]

### Genotypic characterization using whole genome sequencing

Whole genome sequencing (WGS) was carried out on only fifteen isolates due to economic limitations. The isolates were selected to represent MRSP-positive bitch-litter units from different sampling times and successive parturitions of the same bitch (Table 1). A sixteenth isolate dating from 2013, cultured from the milk of a bitch who was again MRSP-positive in 2014–2016, was also included in the WGS analysis [[14]; bitch N° 3].

DNA was extracted from agar-plated colonies using an EZ1 Advanced and EZ1 DNA tissue kit (Qiagen, Hilden, Germany), and DNA concentration was determined using a Qubit HS DNA kit (Life Technologies, Carlsbad, CA, USA). Sequencing libraries were created using the Nextera XT kit (Illumina, San Diego, CA, USA), and 250-bp paired-end sequencing was performed with a MiSeq sequencer (Illumina). Genome assembly was performed with SPAdes v.3.9.1 using the ‘—careful’ parameter [20] and an average coverage of 200×. Whole-genome sequence reads for the 16 isolates were deposited in the European Nucleotide Archive with the study accession number PRJEB19741. The 7-loci sequence types were determined by uploading the assemblies to the pubMLST server [21].

Core genome MLST (cgMLST) was performed using SeqSphere+ v3.5.0 software (Ridom GmbH, Münster, Germany), with cgMLST target genes extracted from the MRSP E140 genome (NCBI Accession: NZ_ANOI). The resulting cgMLST scheme consisted of 2183 genes for phylogenetic analysis (Additional file 1: Table S1). For comparison, unrelated MRSP genomes were added to the analysis: one Danish ST71 (E140), one German ST71 (E104, NCBI accession: LAWU), five genomes representing different ST71-clusters from a Swedish study [22] and five reference isolates, 081661 (NCBI accession: NZ_CP016073), 9,841,787 (NCBI accession: JTKQ), 9,841,998 (NCBI accession: JTKP), 2001–08-299 (NCBI accession: JTKO) and NA45 (NCBI accession: NZ_CP016073). To visualize the number of allelic differences between the genomes, minimum spanning trees were created in SeqSphere+.

Using task templates based on the *S. aureus* microarray (Alere Technologies GmbH, Jena, Germany) available in SeqSphere+, occurrence of genes involved in regulation, resistance and virulence was assessed. Occurrence of genes encoding antibiotic resistance was also verified using the Comprehensive Antibiotic Resistance Database (https://card.mcmaster.ca/, 2017-07-03). In addition, occurrence of the *S. pseudintermedius* toxin genes *luk*I/S (X79188), *siet* (AB099710), *SE-INT* (AB116378), *sec*_Canine_, (U91526), *expA* (AB489850) and *expB* (AB569087) was determined by read mapping using Bowtie2 [23].

## Results

From October 2014 to March 2016, MRSP was isolated from two of five bitches tested in kennel A and from four of eleven bitches tested in kennel B, as well as from their puppies. All the bitches were healthy and no puppy died or showed pathological signs during observation.

In kennel A, bitches A1 and A2 were positive for MRSP in either vaginal or milk swabs. A1 was positive in two successive parturitions, 1 year apart, and had already had MRSP-positive milk after a previous parturition in 2013 [[14]; bitch N° 3]. A1 had eleven puppies in 2014 and ten in 2015/16, and of these, four and six were tested respectively, with all being positive for MRSP at least once in the oral mucosa or abdominal skin (Table 1). A2 had one pup in 2014 and two in 2015/16, and all three were tested and shown to be MRSP-positive at least once (Table 1).

**Fig. 1:**
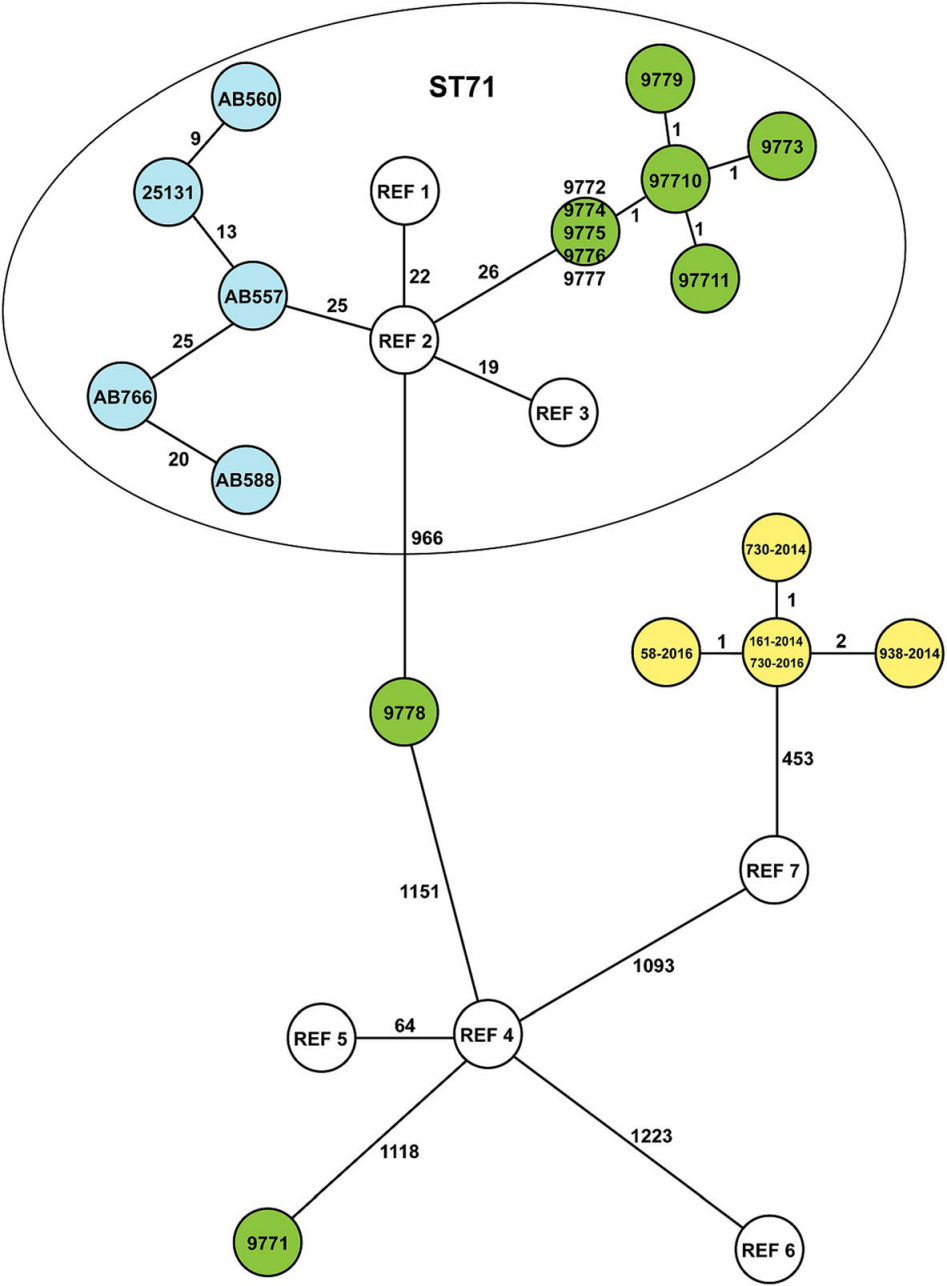
Minimum spanning tree of the core genome MLST results from all 16 MRSP isolates (green for kennel A and yellow for kennel B) and from five Swedish isolates representing five different clusters of ST71 isolates (blue) and reference isolates from GenBank (white). The white isolates comprise strains (with NCBI accessions in parentheses): REF 1: 081661 (NZ_CP016073), REF 2: E104 (LAWU), REF 3: E140 (ANOI), REF 4: 9841787 (JTKQ), REF 5: 9841998 (JTKP), REF 6: 2001–08-299 (JTKO), REF 7: NA45 (NZ_CP016073). The results are based on analysis of 1626 genes. The numbers represent the number of allele differences between isolates, and the lines are not proportional to the number

All *S. pseudintermedius* isolates from kennel A were MRSP, for a total of 22. Of these, ten were selected for WGS. With two exceptions, every isolate belonged to the seven-gene multi-locus sequence type (7-MLST) ST71. One isolate from bitch A1 on day 35, after the last parturition, (Table 1, isolate 9778) belonged to the new type ST688, which shares five of seven alleles with ST71, while the isolate from the same bitch in 2013 (Table 1, isolate 9771) belonged to type ST258. ST258 is distinct from both ST71 and ST688 based on the 7-MLST scheme and the cgMLST (Fig. 1). Based on cgMLST, all ST71 isolates were genetically related, differing only in up to four alleles of the 2100 genes when compared pairwise (data not shown). Isolates from kennel A formed a separate cluster from other MRSP-ST71 genomes available from public databases (Fig. 1).

In kennel B, bitches B1-B4 were MRSP-positive in either vaginal or milk swabs. Two puppies from B1 and B2, from two litters of two, yielded MRSP-positive oral mucosa swabs on day 3 (Table 1). MRSP-positive bitches and puppies also carried non-methicillin-resistant strains.

Of the eight MRSP isolates identified, five were selected for WGS, following the same criteria as for kennel A. The five sequenced isolates all belonged to the new type ST772. Based on cgMLST, these isolates clearly differed from those in kennel A but were less diverse, differing only by 1–7 alleles (see Fig. 1).

Most isolates genotyped from kennel A were resistant to seven antimicrobial classes other than beta-lactams, with susceptibility only to rifampicin (Table 3). Reduced susceptibility to amikacin was shown by six out of nine ST71 isolates (Table 3). The phenotypic resistance profile was confirmed by the presence of resistance genes. The ST71 isolates all carried the methicillin-resistance gene *mecA*, the beta-lactamase gene *blaZ*, and genes encoding resistance to aminoglycosides *aac(6′)-Ie-aph(2″)-Ia*, *aph(3′)-IIIa* and *aad* [6], macrolide *erm(B)*, streptothricin *sat-4* and trimethoprim *dfrG*. All isolates were positive for *tetK,* which encodes for tetracycline resistance, except isolate 97711 from the oral mucosa of a puppy from bitch A2 at 35 days after the second parturition. Based on cgMLST, the 97711 isolate differed by only one allele from isolate 97710, which was isolated simultaneously from bitch A2. The lack of *tetK* was due to the loss of a plasmid virtually identical to the 4.5-kb plasmid pT181 (J01764.1), first described in *S. aureus* [24]. Isolate 97711 was indeed susceptible to doxycycline in vitro*.* The ST688 isolate, that was susceptible to gentamycin and amikacin, lacked the genes *aac(6′)-Ie-aph(2″)-Ia* and *tetK* but carried *tetM* for tetracycline resistance and *cat* for chloramphenicol resistance (Table 3). ST258, dating from 2013, was sensitive to several tested antimicrobials. It showed a similar resistance gene profile to ST688 but lacked *cat* and carried *qac*C, which mediates resistance to antiseptics, disinfectants and beta-lactam antibiotics [25].

**Table 3:** Resistance profile of MRSP genotyped strains of kennel A (*N* = 11)

DOG	A1						A2				
YEAR	2013	2014		2015/16			2014	2015/16			
	b	p	p	b	p	p	p	b	b	b	p
Isolate Identification N°	9771	9776	9775	9778	9774	9772	9777	97710	9779	9773	97711
MLST	ST258	ST71	ST71	ST688	ST71	ST71	ST71	ST71	ST71	ST71	ST71
Antimicrobial agent											
Oxacillin	R	R	R	R	R	R	R	R	R	R	R
Ampicillin	R	R	R	R	R	R	R	R	R	R	R
Penicillin	R	R	R	R	R	R	R	R	R	R	R
Ticarcillin	S	R	R	R	R	R	R	R	R	R	R
Ticarcillin/Clavulanic acid	S	R	R	R	R	R	R	R	R	R	R
Amoxicillin/Clavulanic acid	S	R	R	R	R	R	R	R	R	R	R
Cefazolin	S	R	R	R	R	R	R	R	R	R	R
Cefpodoxime	R	R	R	R	R	R	R	R	R	R	R
Ceftiofur	R	R	R	R	R	R	R	R	R	R	R
Trimethoprim-sulfamethox.	R	R	R	R	R	R	R	R	R	R	R
Gentamicin	S	R	R	S	R	R	R	R	R	R	R
Amikacin	S	S	I	S	I	I	S	I	R	I	I
Rifampicin	S	S	S	S	S	S	S	S	S	S	S
Enrofloxacin	S	R	R	R	R	R	R	R	R	R	R
Marbofloxacin	S	R	R	R	R	R	R	R	R	R	R
Clindamycin	R	R	R	R	R	R	R	R	R	R	R
Erythromycin	R	R	R	R	R	R	R	R	R	R	R
Doxycycline	R	R	R	R	R	R	R	R	R	R	S
Chloramphenicol	I	I	I	R	I	I	I	I	I	I	I

*b* bitch, *p* puppy, *S* susceptible, *I* intermediate, *R* resistant

Similar to the kennel A isolates, all kennel B isolates showed multi-resistance profiles, although they differed in fluoroquinolone susceptibility. Most of them were resistant to both amikacin and rifampicin (Table 4). They were all identical in the genes encoding antibiotic resistance, namely, *aac(6′)-Ie-aph(2″)-Ia*, *aph(3′)-IIIa*, *aad* [6], *dfrG*, *sat-4* and *ermB*.

**Table 4:** Resistance profile of MRSP genotyped strains of kennel B (*N* = 5)

DOG	B1		B2		
	p	b	b	p	b
Isolate Identification N°	58–2016	161–2014	730–2016	730–2014	938–2014
MLST	ST772	ST772	ST772	ST772	ST772
Antimicrobial agent					
Oxacillin	R	R	R	R	R
Ampicillin	R	R	R	R	R
Penicillin	R	R	R	R	R
Ticarcillin	I	S	S	R	I
Ticarcillin/Clavulanic acid	S	S	S	R	R
Amoxicillin/Clavulanic acid	S	R	R	R	R
Cefazolin	R	R	R	R	R
Cefpodoxime	R	R	R	R	R
Ceftiofur	R	R	R	R	R
Trimethoprim-sulfamethox.	R	R	R	R	R
Gentamicin	R	R	R	R	R
Amikacin	R	R	R	R	R
Rifampicin	R	S	S	R	R
Enrofloxacin	S	S	S	R	R
Marbofloxacin	S	S	S	R	R
Clindamycin	R	R	R	R	R
Erythromycin	R	R	R	R	R
Doxycycline	R	S	R	R	R
hloramphenicol	R	I	R	R	R

*b* bitch, *p* puppy, *S* susceptible, *I* intermediate, *R* resistant

All MRSP isolates from kennels A and B contained the toxin genes *luk*I/S, *siet* and SE-INT.

## Discussion

WGS revealed two different situations in the investigated kennels. In kennel A, multiple strains continued to circulate, as previously evidenced by PFGE in 2013 [14], while in kennel B, the isolates were highly related, with a new ST772 type circulating. A possible explanation for the difference between the two kennels could be that the introduction and spread of MRSP in kennel B was more recent or that the selection pressure was weaker, as suggested by the concomitant isolation of MSSP and MRSP from the bitches and puppies of kennel B. Both kennels represent two distinct settings with different factors, among which could be the amount and type of antimicrobial use, which may have influenced the selection and spread of the different MRSP strains. Although beta-lactams are the first-choice antimicrobials for treating breeding bitches in both investigated kennels, we have no precise data on the general frequency/dose/reasons that these agents were administered.

MRSP isolates are commonly multi-drug resistant, but resistance to amikacin and rifampicin is an emerging trait. Amikacin-resistant strains have been isolated with increasing frequency from canine patients [26] but not from healthy dogs. In *S. pseudintermedius*, the most common amikacin resistance gene was found to be *aph(3′)-IIIa* [26], which is present in the isolates detected here. Aminoglycosides such as amikacin are among the last choices of treatment for staphylococcal infections and have not been used on the bitches included in this study. In addition, rifampicin resistance, due to mutation in the *rpoB* gene, is reported in MRSP isolated from infected dogs treated with rifampicin [27], which is not the case with the healthy breeding bitches in this study.

The detection of the gene *qac*C in the isolate dating from 2013 is also remarkable. This plasmid-borne gene, responsible for transporting quaternary ammonium compounds and ethidium bromide out of the cell, has been detected in *S. aureus* and coagulase-negative staphylococci but not in *S. pseudintermedius* [28]*.* Quaternary ammonium disinfectants are commonly used in kennels, and repeated sub-lethal exposure could have selected for the expression of this efflux pump. However, a gene-transfer from other *Staphylococcus* species could also have been a possible explanation for the presence of qacC [28].

Dogs with clinical MRSP infections can be carriers for several months after acquisition [29, 30], but data on MRSP colonization persistence and maternal-offspring transmission are scant. Previous studies have reported that individual dogs can be either persistent or intermittent carriers of highly genetically diverse MRSP strains, isolated from different body sites [1]. We therefore cannot exclude the possibility of the simultaneous presence of different STs in the bitch that carried ST258 in 2013 [14] and ST688 in 2014. Moreover, ST71 was isolated from her puppies; although we could hypothesize an environmental origin for ST71, the maternal source is also possible. The chosen sampling locations may have influenced these results, and testing different or additional body sites could have yielded more MRSP genotypes on a single animal at the same sampling time [30].

In our work, MRSP was only sporadically isolated from mothers and puppies during the 35-day sampling period. Furthermore, MRSP was not always present simultaneously in the bitches and their puppies. Interestingly, however, we showed that MRSP occurrence in the puppies appeared to be connected to MRSP-positive bitches. Apart from two exceptions in kennel B, MRSP was isolated at different times from all litters of the colonized bitches and from most of the tested puppies from the colonized litters. A potential transmission from bitch to offspring, or at least a spread within the kennel, appears to be likely due to the high genetic relatedness of the strains within a kennel. Previous studies have shown that puppies are colonized with *S. pseudintermedius* almost immediately after birth and that the first contact is from the buccal flora of their mother because the amniotic membrane is generally intact when puppies are born [2, 31]. Furthermore, close contact during nursing contributes to the rise of cutaneous *S. pseudintermedius* flora in puppies [31]. Another likely source for the colonization of puppies is the milk and the mammary glands. *S. pseudintermedius* is commonly isolated from the milk of healthy post-partum bitches due to the sinus system of the gland having been colonized [32, 33]. In the current study we cannot exclude the milk having been contaminated by the skin during sample collection because samples were milked cumulatively from every mammary gland.

## Conclusions

Our data show that MRSP circulation can differ in different settings, from the simultaneous detection of multiple strains to the isolation of a single genotype, such as with the case of the new ST772 type.

Worth noticing is the detection of amikacin- and rifampicin-resistant isolates carried by healthy bitches and puppies that had not been treated with these antimicrobials.

Vertical bitch-litter transmission appears common since MRSP was isolated at different sampling times from most of the litters of the colonized bitches and from most puppies tested from the colonized litters. It is also likely that the bitches were the primary source since the MRSP from the puppies was only isolated from litters of MRSP-positive bitches.

Our study only covered the period from birth to weaning; thus, it would be interesting to assess how long the MRSP is carried, whether the bitches are colonized in different locations during and after the end of lactation, and how different locations may affect potential transmission to the puppies.

## Additional file

Additional file 1: **Table S1.** (XLS 260 kb)
